# Electronic angle focusing for neutron time-of-flight powder diffractometers

**DOI:** 10.1107/S1600576724008756

**Published:** 2024-10-01

**Authors:** Robert B. Von Dreele

**Affiliations:** a Advanced Photon Source Argonne National Laboratory 9700 South Cass Avenue Lemont IL60439-4814 USA; Australian Nuclear Science and Technology Organisation, Lucas Heights, Australia

**Keywords:** neutron powder diffraction, electronic focusing, profile shape functions, time of flight

## Abstract

A neutron time-of-flight (TOF) powder diffractometer with a continuous wide-angle array of detectors can be electronically focused to make a single pseudo-constant wavelength diffraction pattern, thus facilitating angle-dependent intensity corrections. The resulting powder diffraction peak profiles are affected by the neutron source emission profile and resemble the function currently used for TOF diffraction.

## Abstract 2

A wide-angle short-pulse spallation neutron detector bank is shown to provide an opportunity for data processing to make a pseudo-constant wavelength powder diffraction pattern.

## 1. Introduction

The earliest powder diffractometers constructed for a neutron spallation source achieved focusing by arranging detectors in panels tilted with respect to the nominal scattering angle so that all neutrons scattered by a given *d* spacing across the face of the detector panel had the same time of flight (TOF) (Jorgensen & Rotella, 1982 ▸). This ‘geometric time focusing’, while useful at backscattering angles, was not practical at lower angles because of the extreme tilts required for focusing. Electronic time focusing (Crawford *et al.*, 1981 ▸) allowed other geometries with greater detector coverage of solid angle to be used. For example, the 144 detectors (10 atm ^3^He tubes) for the general-purpose powder diffractometer (GPPD) (Faber & Hitterman, 1985 ▸) at IPNS (at Argonne National Laboratory, in operation 1981–2008) were placed on a ring 1 m from the sample position; they were grouped into ten ‘banks’ at various scattering angles symmetrically disposed about the incident beam. Given the de Broglie relation between neutron velocity and wavelength and Bragg’s law, electronic time focusing is then carried out for each neutron detector event from its TOF (*T*) and the instrument geometry [equation (1)] to make a pseudo-TOF (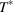) that puts all neutrons scattered from the same *d* spacing into the same TOF bin:
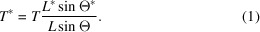
Each detector element (tube or scintillator pixel) has a flight path (*L* = source-to-sample plus sample-to-detector-element distance) and scattering angle (2Θ), as determined by the construction details of the instrument; a bank of detector elements is assigned a global scattering angle (2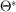) and distance (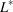) for use in equation (1). From the de Broglie relation, these terms can be used to calculate the mean wavelength (λ in Ångstrom) needed for certain intensity corrections (absorption, extinction and neutron resonance scattering) for each Bragg peak:
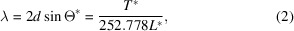
for 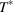 in microseconds and 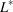 in metres. Because a detector bank spans a range of scattering angles, these equations imply that a range of neutron wavelengths will contribute to each Bragg peak; an estimate of this range based on the angle derivative of Bragg’s law is
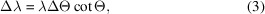
where ΔΘ is in radians and is the angular span of a detector bank. This wavelength band is strongly angle dependent. For example, while a backscattering detector bank (2Θ = 140°, ΔΘ = 10° and λ = 1.5 Å) has a wavelength spread of only ∼0.05 Å, the same detector bank at 2Θ = 20° has a wavelength spread of ∼0.4 Å, which could compromise wavelength-dependent intensity corrections for such low-angle banks. A similar bank at 2Θ = 60° gives a more tolerable Δλ ≃ 0.2 Å.

Spallation neutron sources operate at a particular frequency of pulse repetition, typically 20–60 Hz. Thus, the available TOF range is limited by this frame rate; this imposes a maximum neutron wavelength for a TOF diffractometer with a given total flight path via equation (2). The flight path length is restricted by the need to avoid contamination of the diffraction pattern by neutrons from the tail of the neutron emission spectrum in the previous frame (‘frame overlap’) and the prompt neutrons from the next frame. The long-wavelength part of the neutron emission spectrum follows Maxwell–Boltzmann statistics for typical moderators, and the neutron intensity for λ > 5 Å is negligible. Thus, from equation (2), the maximum instrument length should be ∼20 m to avoid frame overlap for a 30 Hz source (*e.g.* IPNS); the GPPD was built to this specification.

The resolution of a neutron TOF diffractometer can be estimated as a sum of the variances of the contributions to a diffraction line-width variance:
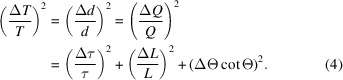
For the second line of the equation, the first term arises from the distribution of emission times from the source moderator for neutrons of a particular wavelength; this is asymmetric with a fast rise followed by a slow decay. The second term is from the distribution in neutron path lengths including the start location within the moderator, the position within the sample volume for scattering and the point in the individual detector tube/pixel where the neutron is detected. The last term is from the angular spread in the scattering events for these neutrons. Each bank of detectors can be tilted to make the sum of the second and third terms uniform across the bank (‘resolution focusing’). Then, to maximize intensity, instrument design usually attempts to match these three terms in backscattering detectors; banks of detectors at lower angles are then positioned further from the sample to partially compensate for the broadening from the third term.

Numerous neutron TOF powder diffractometers have been constructed following these principles; they all feature multiple detectors (tubes or scintillators) grouped into banks at a small selection of scattering angles. For example, the GEM diffractometer (Williams *et al.*, 1997 ▸) has the sample 18.7 m from the 50 Hz ISIS source with seven banks of detectors. A *T*-zero (*T*_0_) chopper removes the prompt neutron and gamma-ray pulse from the spallation target when it is struck by the proton beam. This chopper is open when the useful thermal neutrons have appeared from the moderator. The wavelength range is ∼0.4–3.2 Å and the peak-width resolution (Δ*T*/*T*) is ∼0.007 for the backscattering detector bank. The HRPD instrument at ISIS (Johnson & David, 1985 ▸; Ibberson, 2009 ▸) has a 98 m curved supermirror guide that puts the sample position out of the line of sight of the source, thus shielding it from the prompt pulse and giving a 10–100× increase in neutron flux on the sample. There are four banks of detectors: one backscattering (Δ*T*/*T* ≃ 0.001), two at 90° 2Θ on opposite sides of the sample and one at low angles (∼30° 2Θ). Choppers are used to remove four of five pulses resulting in an effective 10 Hz operation, so the wavelength range is ∼0.40–4.4 Å (0.2 < *d* < 2.2 Å for the backscattering detector bank). A single 50 Hz pulse wavelength frame would only be ∼0.8 Å wide, which is insufficient for useful TOF diffraction. All these instruments produce multiple datasets, each covering different ranges of *d* spacing according to their assigned scattering angles. Consequently, numerous Rietveld (1969 ▸) refinement computer programs have been developed to refine a crystal structure with multiple datasets [*e.g.GSAS* by Larson & Von Dreele (2004 ▸) and *GSAS-II* by Toby & Von Dreele (2013 ▸)]. The assigned scattering angles for each detector bank are sufficient for making angle-dependent intensity corrections for texture (Von Dreele, 1997 ▸), absorption (Lobanov & Alte da Veiga, 1998 ▸; Larson & Von Dreele, 2004 ▸) and extinction (Sabine *et al.*, 1988 ▸).

**Figure 1:**
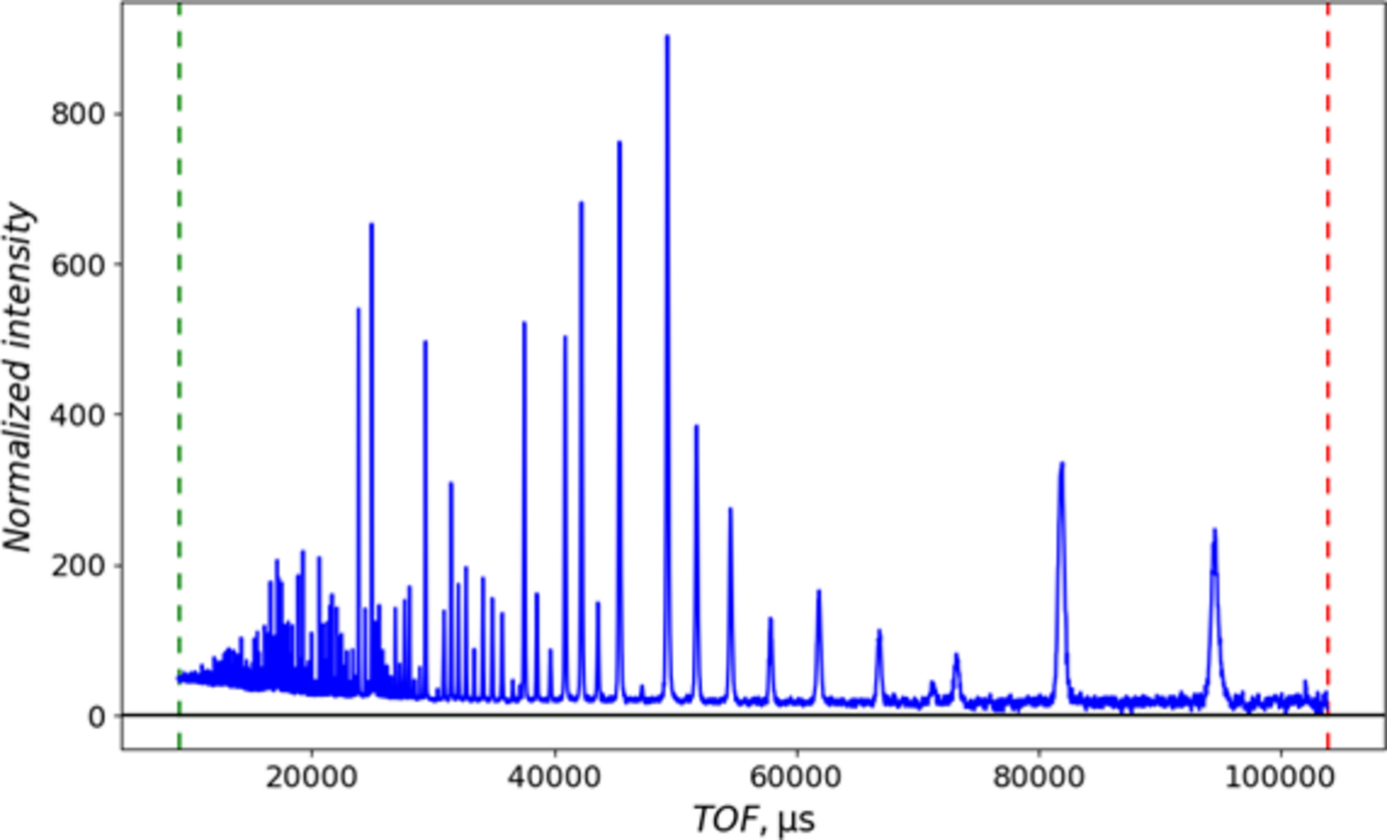
A neutron TOF pattern of cubic Ca_3_Al_2_Na_2_F_14_ (‘NAC’, *I*2_1_3, *a* = 5.46 Å; minor phase CaF_2_ also present) from POWGEN (SNS, Oak Ridge National Laboratory) for the 0.566 < λ < 1.566 Å band (2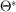 = 90°, 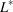 = 63.18 m); this covers 0.4 < *d* < 4.6 Å for 9040 < 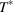 < 103 800 µs.

The POWGEN diffractometer (Huq *et al.*, 2011 ▸, 2019 ▸) at SNS (Oak Ridge National Laboratory) represents a departure from the conventional multibank TOF diffractometer layout. The 40 scintillator detector panels cover the surface of an equiangular spiral cylinder in 12 columns on both sides of the instrument beam axis. The angular coverage is 10 < 2Θ < 170° in the horizontal plane (24 panels), and the remaining 16 panels are positioned above and below on one side of the instrument. Sample-to-detector-panel distances vary from 2.5 m in backscattering to 4.7 m at the lowest angles. The panels are mounted to minimize gaps, so the angle coverage is essentially complete over 1.2 steradians (12 m^2^). Thus, the 43 120 pixels are each characterized by individual 2Θ and flight paths to allow application of equation (1) (with 2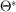 = 90° and 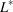 = 63.18 m), forming a pseudo-TOF (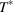) for each pixel to generate a single powder diffraction pattern that encompasses neutron events across the entire suite of detectors (Fig. 1 ▸). This data processing includes corrections for empty cans/empty instruments as well as normalization by a vanadium spectrum according to an algorithm outlined by Huq *et al.* (2019 ▸). POWGEN is equipped with a *T*_0_ chopper and frame choppers that define a wavelength band (1 Å wide, as limited by 60 Hz SNS operation and 63–65 m total flight path) offset from *T*_0_. Current operation typically centres this wavelength band at 0.8, 1.5 or 2.665 Å for an individual data-collection run; other settings are readily available (*cf*. Fig. 1 ▸) but must be accompanied by the necessary standard calibration spectra required for data processing.

Clearly, the angle associated with each scattered neutron is lost for POWGEN TOF data. For example, a diffraction peak at *d* = 1.5 Å (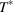 = 33 900 µs) has neutron events in detectors with 39 < 2Θ < 84° for the band 1 < λ < 2 Å centred at 1.5 Å; the assigned 2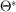 = 90° for POWGEN is clearly incorrect. Thus, angle-dependent intensity corrections cannot be used from within a Rietveld refinement, unlike multibank TOF diffractometers where the angular width of a detector bank is ∼10° 2Θ. The neutron-absorption correction is usually proportional to wavelength, which is typically obtained in a Rietveld code by use of equation (2) from the TOF and the assigned 2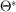 or 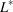. For POWGEN, this can be seriously incorrect; for example, the first peak for Al_2_O_3_ at *d* = 3.48 Å is found at 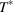 = 78 575 µs, which implies λ = 4.92 Å via equation (2), whereas the actual neutron wavelengths are in the band 1 < λ < 2 Å. Consequently, data from POWGEN cannot be corrected for texture effects, absorption or extinction via the Rietveld method. Absorption corrections can be applied during data processing on the basis of sample composition and a transmission measurement; extinction is rarely encountered, except in highly annealed samples. Textured samples must be avoided for POWGEN experiments.

**Figure 2:**
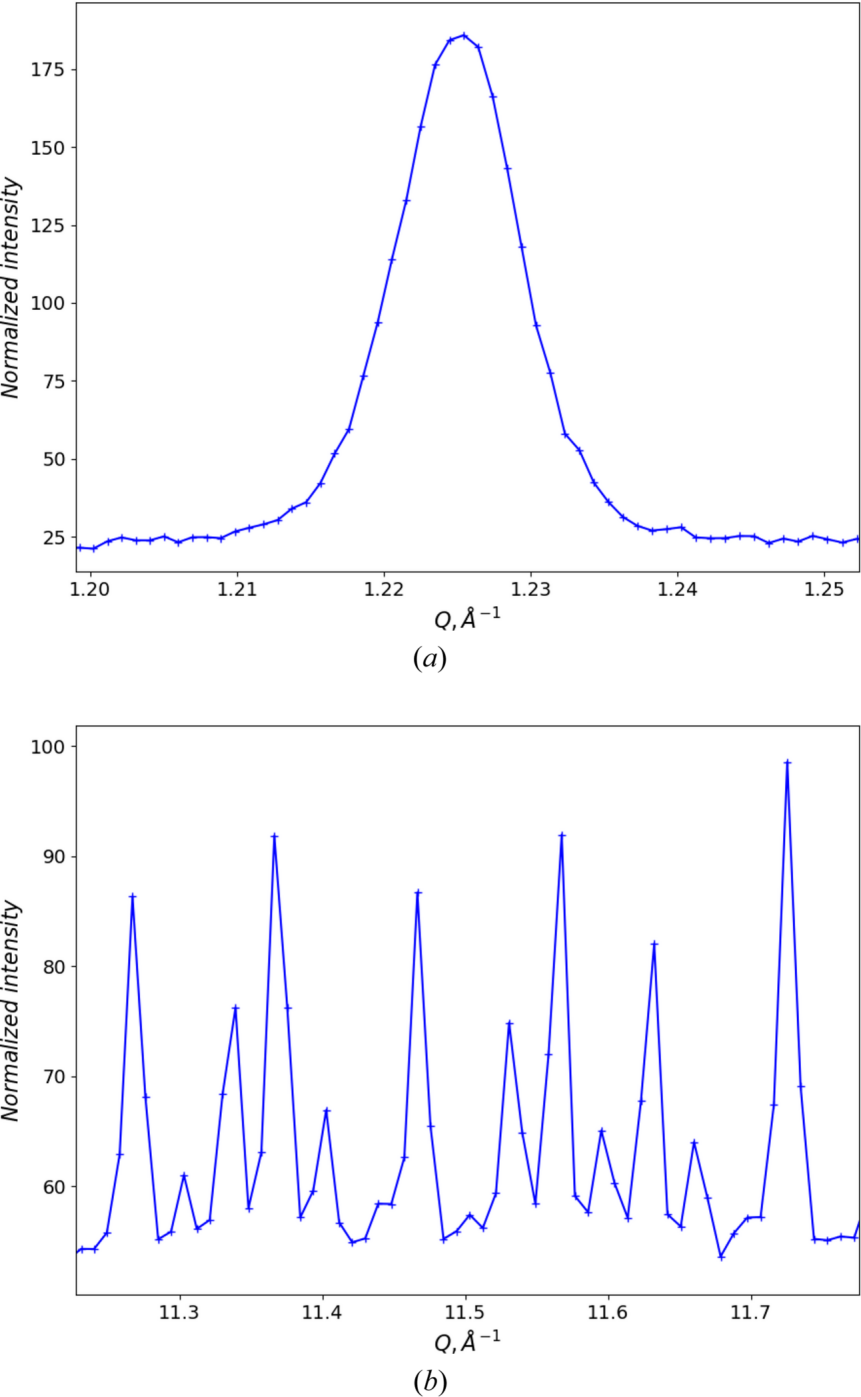
Details of the neutron TOF pattern of cubic Ca_3_Al_2_Na_2_F_14_ (‘NAC’, space group *I*2_1_3, *a* = 5.46 Å; minor phase CaF_2_ also present) from POWGEN (SNS, Oak Ridge National Laboratory) for the 1.0 < λ < 2.0 Å band (2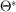 = 90°, 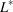 = 63.18 m) plotted as *Q* = 2π/*d* = 252.816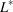 sin 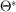/π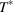. (*a*) A diffraction peak at low *Q* (*d* = 5.126 Å, 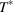 = 115 570 µs) and (*b*) 14 diffraction peaks at high *Q* (*d* ≃ 0.5 Å, 12 100 < 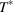 < 12 600 µs). The ‘+’ marks are the observed data points and the curves are guides to the eye connecting them.

The POWGEN resolution (Huq *et al.*, 2019 ▸) follows a smooth parabolic curve, like what is found for typical constant wavelength (CW) powder diffractometers. This is not the same as a multibank TOF instrument, where each bank displays essentially constant Δ*T*/*T* resolution. Consequently, the binning scheme of constant Δ*T*/*T* steps (this is also known as ‘logarithmic binning’), as normally used for TOF data, for POWGEN data leads to an imbalance in the number of data points across a diffraction peak (Fig. 2 ▸). While the number of points is sufficient at low *Q*/large *d* spacing [McCusker *et al.* (1999 ▸) recommend that there should be 6–10 points across the full peak width at half-maximum (FWHM)], it is clearly insufficient at high *Q*/small *d* spacing, which could compromise Rietveld refinement of both peak-shape-sensitive parameters and structural parameters that depend on quality high-resolution data at high *Q*.

To address some of these issues, Jacobs *et al.* (2015 ▸, 2017 ▸) proposed a two-dimensional (2Θ and wavelength dispersive) Rietveld refinement approach in which the neutron events are binned across both 2Θ and wavelength, and then fitted with a two-dimensional profile function and a crystal structure. In that work, long count times were required to get sufficient statistics over the numerous data bins (100K to 1M) needed for the refinement. For the large number of data points, computation times for the refinements were 10–100 times longer than the corresponding one-dimensional ones. Moreover, the profile functions were simplified by leaving out the back-to-back exponentials; this may be appropriate for instruments with pulse-shaping choppers [*e.g.* POWTEX at the FRM II neutron source (Houben *et al.*, 2023 ▸)] but probably not for short-pulse spallation sources such as SNS or ISIS.

As a simpler approach, we propose here an alternative data-binning scheme to give a pseudo-2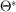 instead of a pseudo-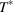 for each neutron event via a rearrangement of equation (1) to give a CW pattern that more naturally follows the detector layout for POWGEN. Unlike conventional CW powder patterns, the peak profiles have an asymmetric shape arising from the spallation neutron pulse; this peak-shape function and its angular dependence are also described with a few examples of its use.

## 2. Data processing

**Figure 3:**
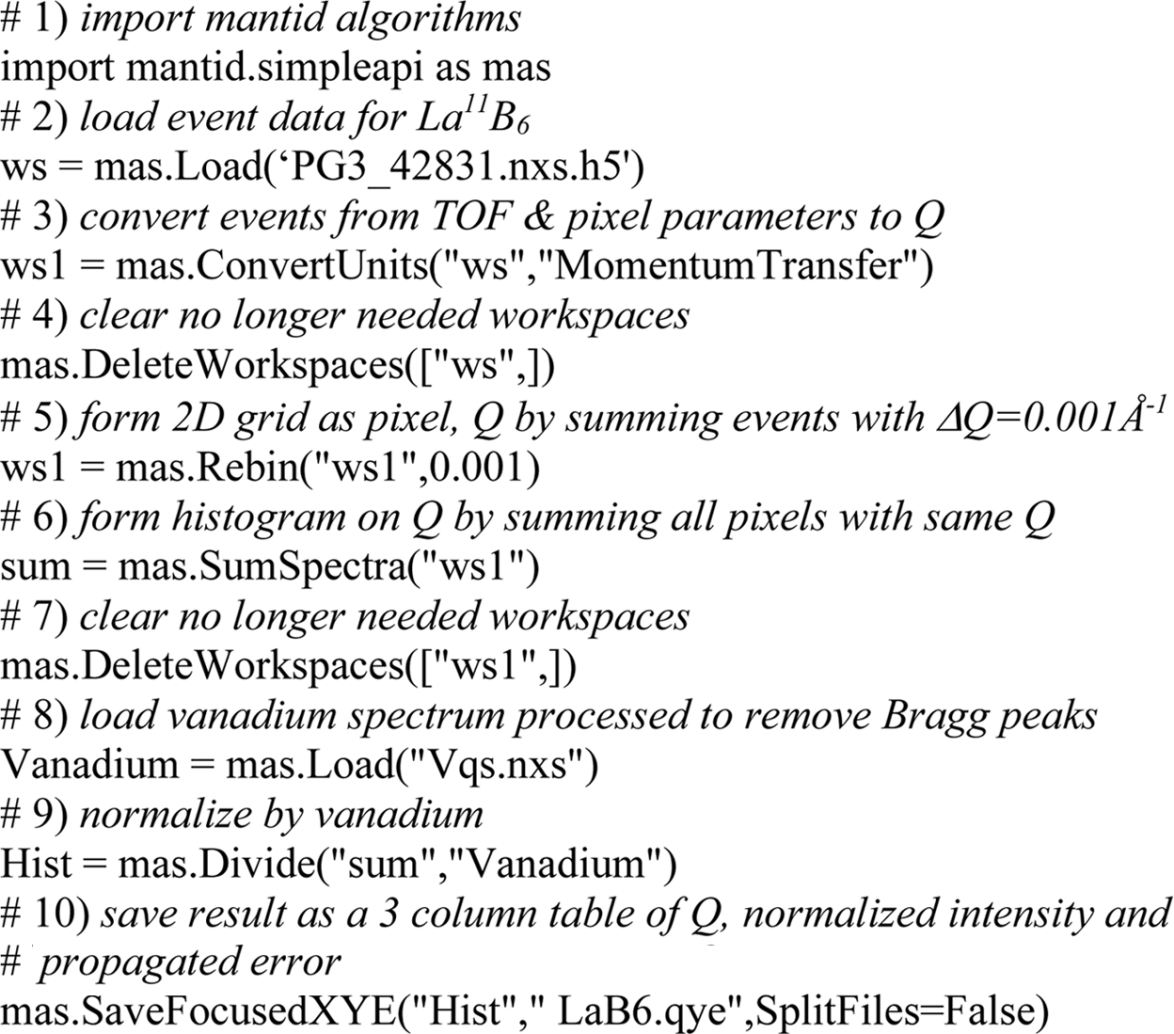
*Mantid* Python script for conversion to *Q*, binning on Δ*Q* = 0.001 Å^−1^ steps, summing and division by the vanadium spectrum to give a normalized neutron powder pattern in *Q* for NIST SRM 660c La^11^B_6_ data collected on POWGEN. Each step is commented in detail.

**Figure 4:**
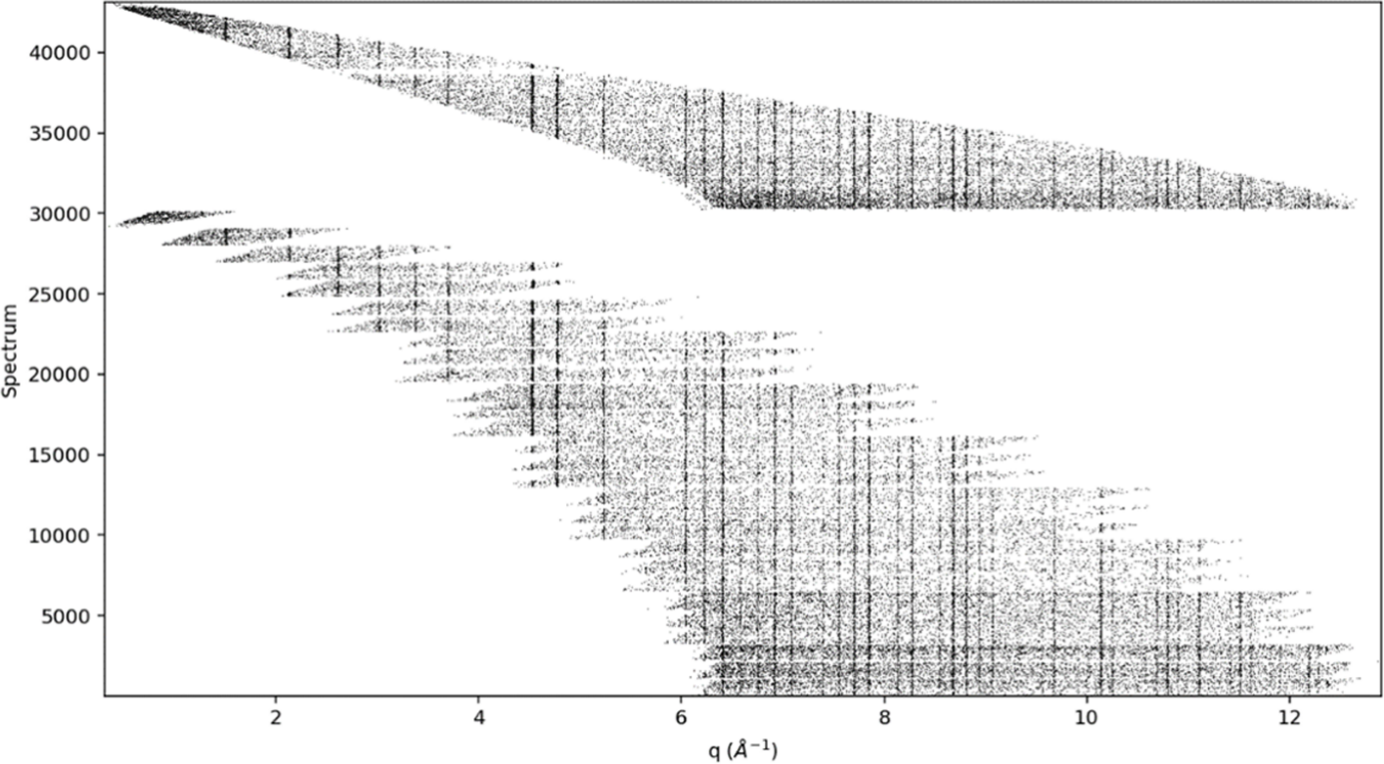
The individual 43 120 pixel spectra accumulated in Δ*Q* = 0.001 Å^−1^ steps from the transformed (*T* → *Q*) event data from POWGEN for NIST SRM 660c La^11^B_6_ from the 1.0 < l < 2.0 Å band plotted as log(*I*). The lowest-angle pixels are to the left in this plot; the backscattering pixels are to the right. The lower block of spectra (<30 000) is from the 28-detector side of the instrument horizontal plane; the upper block is from the 12-detector side. The pixel spectra in the upper block are in order of increasing azimuth, while those in the lower block are numbered in reverse azimuthal order across each detector panel and grouped by increasing 2θ. Dark vertical lines are Bragg peaks as they occur in each pixel spectrum.

**Figure 5:**
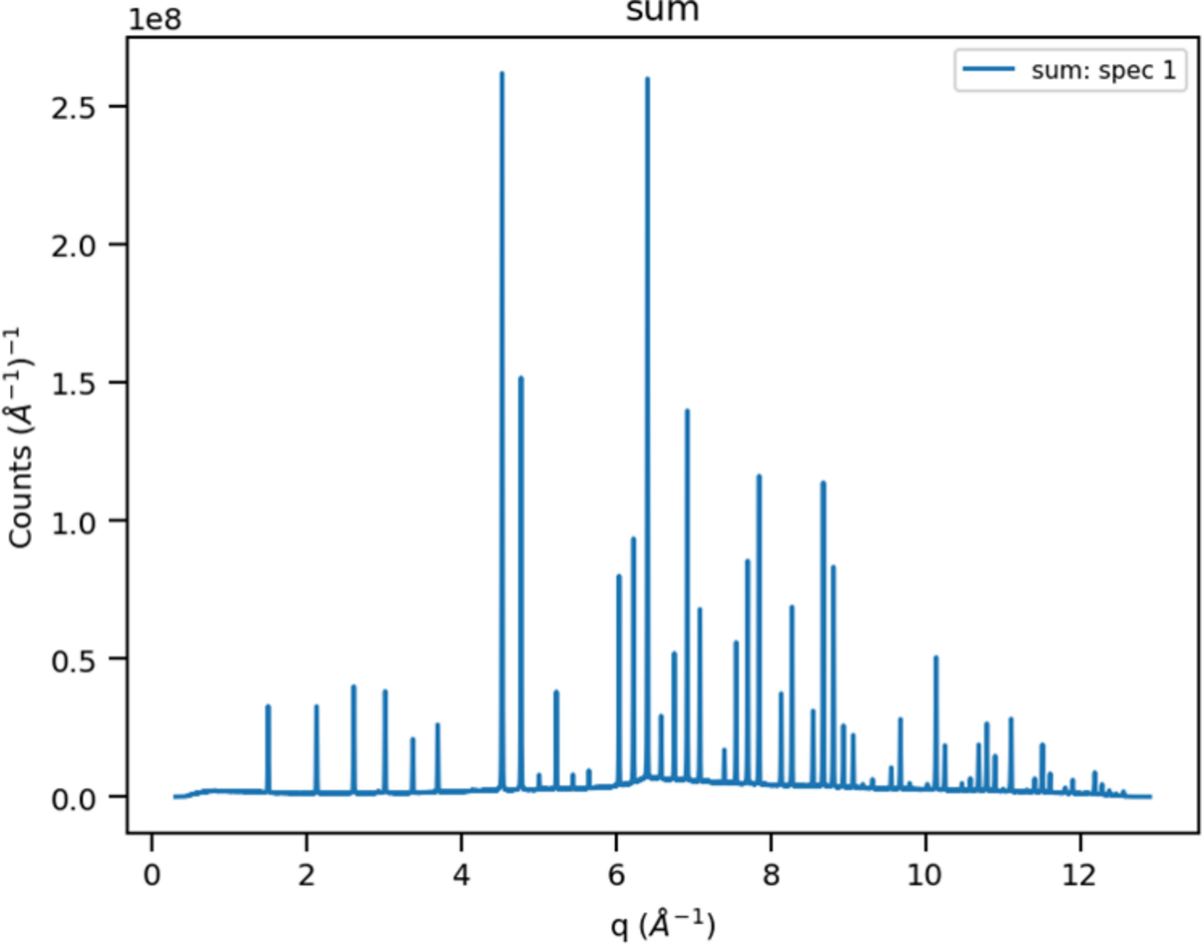
The sum of the 43 120 spectra into ∼13 000 Δ*Q* = 0.001 Å^−1^ bins covering 0.316 < *Q* < 12.899 Å^−1^ for POWGEN data on NIST SRM 660c La^11^B_6_ from the 1.0 < λ < 2.0 Å band.

**Figure 6:**
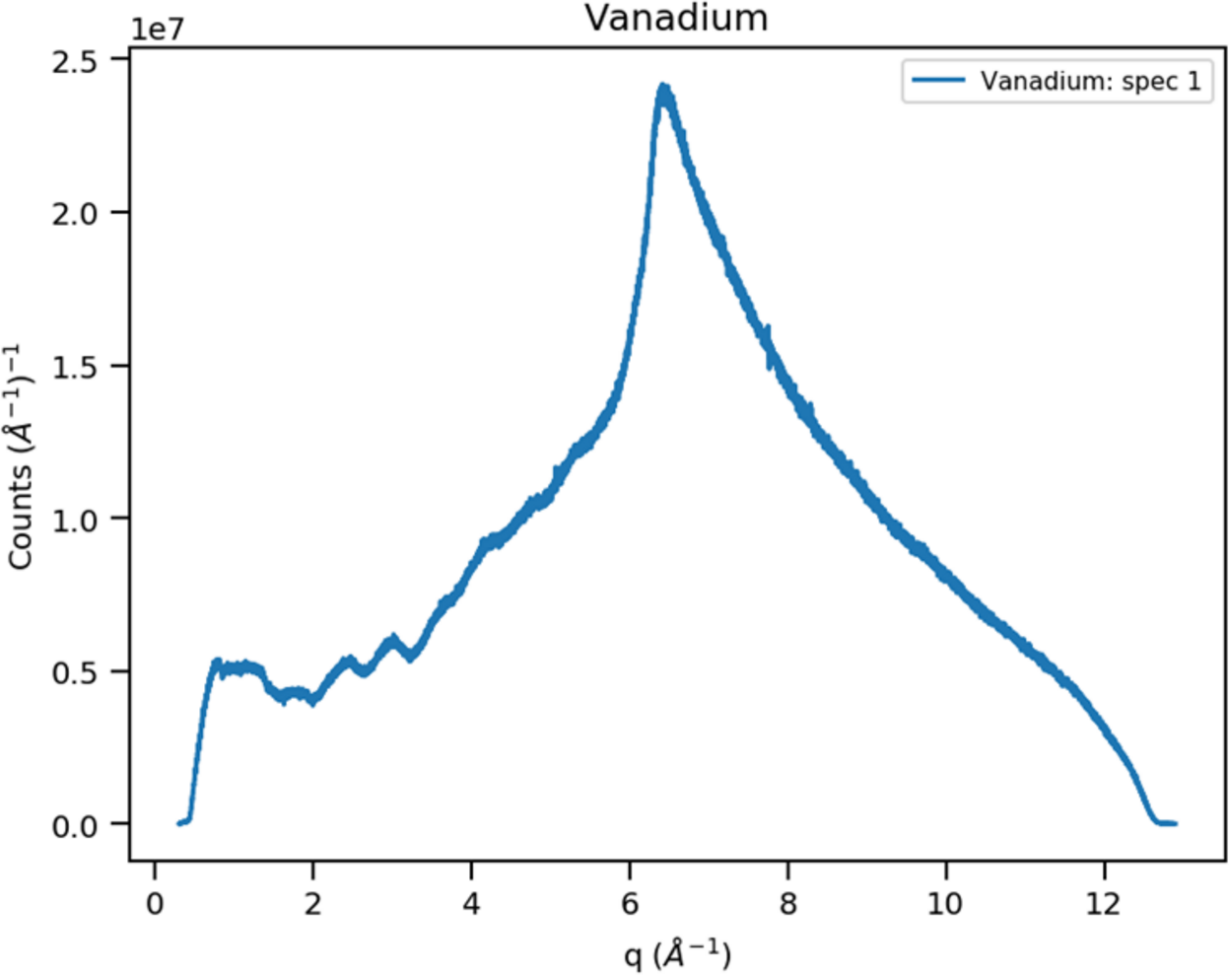
The sum of the 43 120 spectra into ∼13 000 Δ*Q* = 0.001 Å^−1^ bins covering 0.316 < *Q* < 12.899 Å^−1^ for POWGEN data from a vanadium rod for the 1.0 < λ < 2.0 Å band. Vanadium Bragg peaks have been removed.

**Figure 7:**
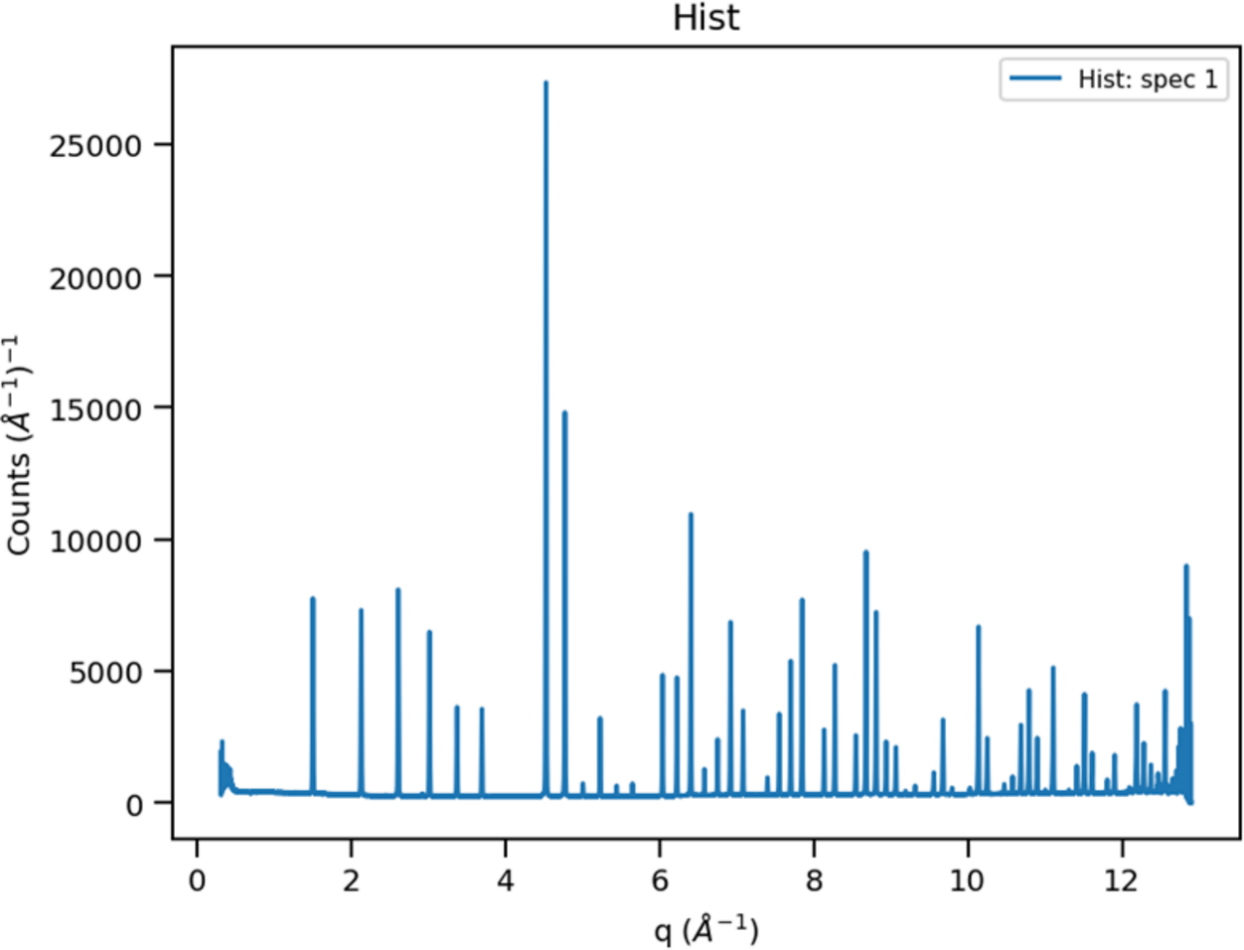
A powder pattern of NIST SRM 660c La^11^B_6_ from POWGEN as normalized by the vanadium pattern in constant Δ*Q* = 0.001 Å^−1^ bins covering 0.316 < *Q* < 12.899 Å^−1^.

The raw datafiles from POWGEN consist of millions of individual neutron detection events (Peterson *et al.*, 2015 ▸) and thus are easily reprocessed by the new algorithm given here; the *Mantid* (Arnold *et al.*, 2014 ▸) data-processing software is used to do this via short Python scripts (*e.g.* Fig. 3 ▸). For step #3 in this figure, each event in the loaded file has a TOF and a detector pixel tag. The latter is used to get the instrument pixel geometry factor, 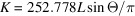, needed to convert TOF to *Q* via a combination of Bragg’s law and the de Broglie equation:
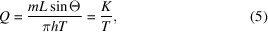
where *m* and *h* are the neutron mass and Planck’s constant, respectively. In step #5, the *Rebin* method distributes the *Q*-based events into 43 120 spectra, one for each detector pixel, covering the approximate range 0.3 < *Q* < 12.9 Å^−1^ in Δ*Q* = 0.001 Å^−1^ steps; the result is shown in Fig. 4 ▸. These are summed in step #6 along *Q* to produce a single powder pattern (Fig. 5 ▸). Based on the appearance of Fig. 4 ▸, clearly the Δ*Q* bins have varying numbers of contributing spectra. Moreover, each pixel subtends a different solid angle depending on its orientation and distance from the sample. In addition, the incident neutron spectrum varies across the wavelength band. All these effects are also present in data for vanadium; the vanadium event data must be collected under the same operational conditions (*e.g.* wavelength span and detector configuration) for any sample runs that would need this vanadium normalization. The vanadium spectrum (Fig. 6 ▸) is processed in the same way as that of La^11^B_6_, but one additional step is removal of the small vanadium Bragg peaks via the *Mantid* method *StripVanadiumPeaks*. Preparation of the vanadium spectrum is described in Section S1 of the supporting information. Dividing the sample spectrum by the vanadium spectrum (step #9) removes all incident spectrum and instrument-geometry effects giving a normalized powder pattern (Fig. 7 ▸) in constant Δ*Q* = 0.001 Å^−1^ steps covering the range 0.3 < *Q* < 12.9 Å^−1^. The number of pixels contributing to each bin in this pattern varies from nearly zero at the extreme ends to 1000s of pixels through the middle part (*cf*. Fig. 4 ▸). This accounts for the poor statistics at *Q* ≃ 0.3 Å^−1^ and *Q* ≃ 12.9 Å^−1^. The last step in the Python script (step #10) writes a datafile with three columns: *Q*, intensity and the estimated standard error in the intensity based on a propagation of errors through the previous steps.

**Figure 8:**
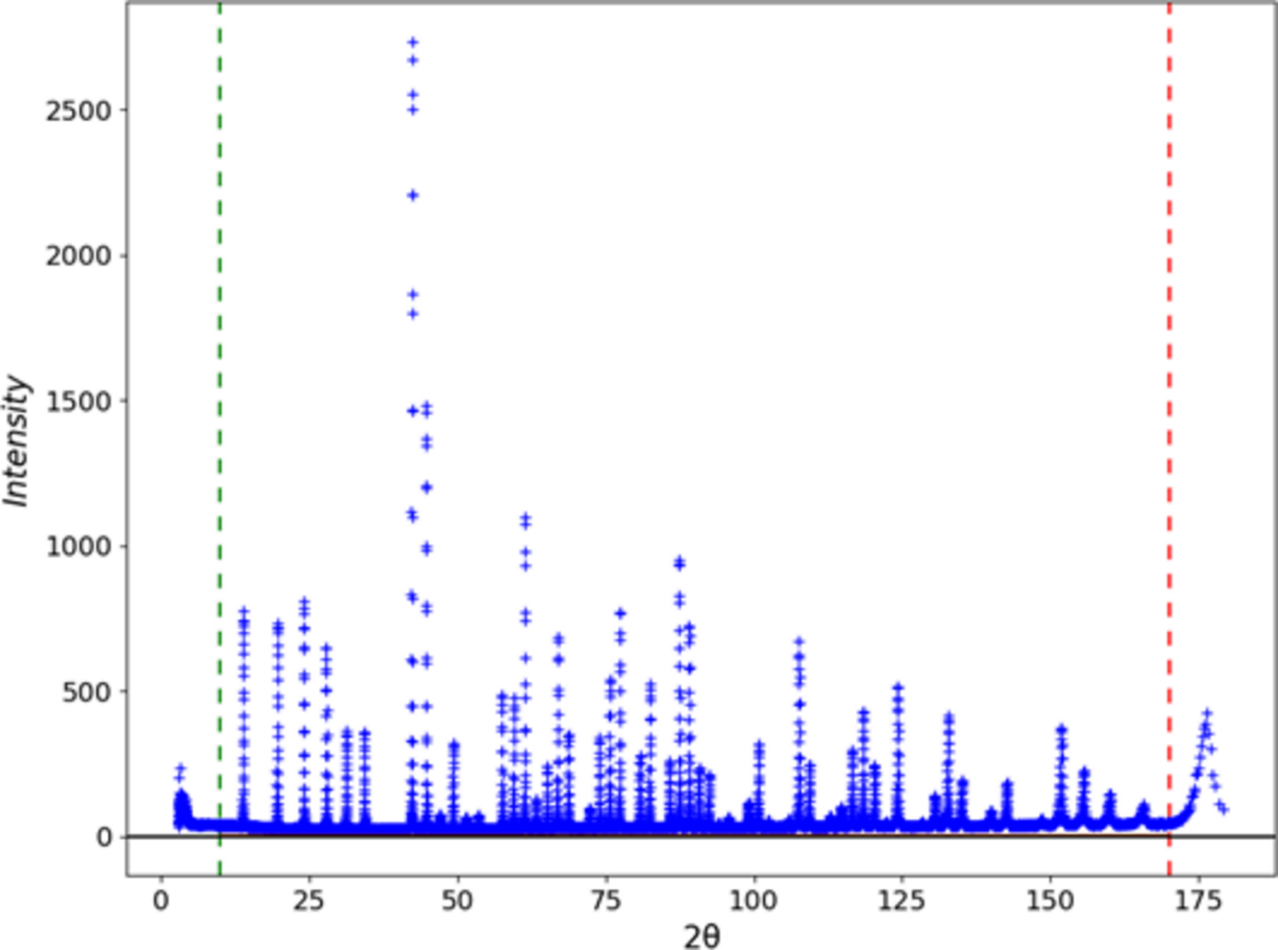
A pseudo-constant wavelength powder pattern of NIST SRM 660c La^11^B_6_ from POWGEN as converted to 2Θ for λ = 1.0 Å upon import into *GSAS-II*. Green and red vertical dashed lines mark the usable range 10 < 2Θ < 170°.

**Figure 9:**
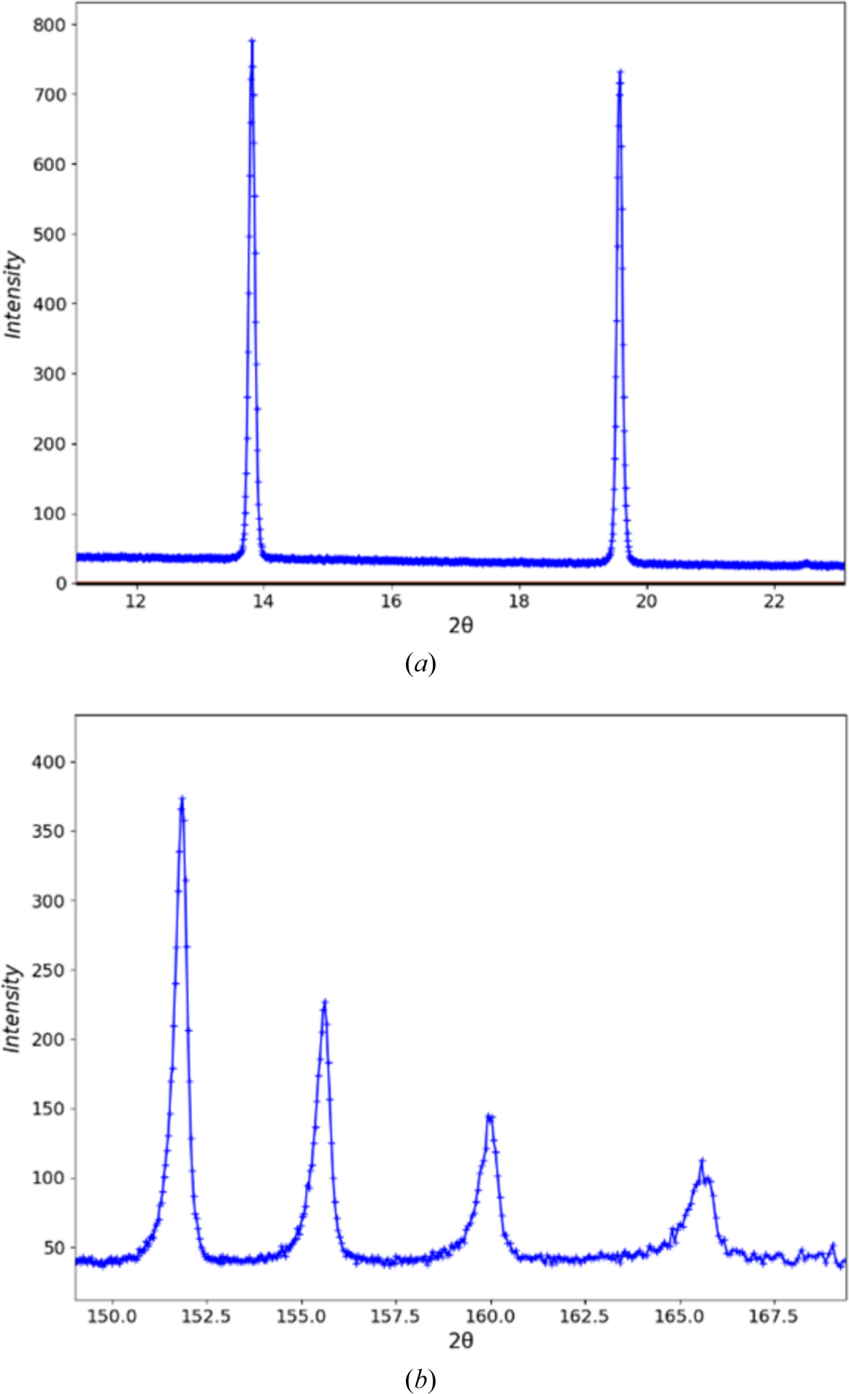
Details of the pseudo-constant wavelength powder pattern of NIST SRM 660c La^11^B_6_ from POWGEN converted to 2Θ for λ = 1.0 Å upon import into *GSAS-II*. (*a*) Two diffraction peaks at low 2Θ and (*b*) four diffraction peaks at high 2Θ. The ‘+’ marks are the observed data points and the curves are guides to the eye connecting them.

Rietveld refinement programs (*e.g. GSAS-II*) work with either 2Θ (CW) or TOF diffraction data; here, we are using 2Θ as it is a close geometric match to the instrumental layout of POWGEN. The conversion requires the selection of a reference wavelength:
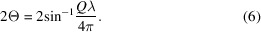
To make use of the available *Q* range and spread it across the angular range subtended by the detectors on POWGEN (10 < 2Θ < 170°), we have selected the wavelength band minimum (λ = 1.0 Å). Equation (6) is applied to the imported data *Q* values within a *GSAS-II* powder data import routine to make a CW representation of the POWGEN data (Fig. 8 ▸) that appears to cover 2.9 < 2Θ < 179.2°; we can select the instrumental range (10 < 2Θ < 170°) as the usable part of the data. Section S2 gives details for use of these data in *GSAS-II*. Fig. 9 ▸ shows that low-angle (low *Q*) and high-angle (high *Q*) diffraction peaks have approximately the same number of steps across their respective FWHMs, which is more than sufficient to satisfy the McCusker *et al.* (1999 ▸) criterion. Choosing a longer wavelength (*e.g.* the band centre, λ = 1.5 Å) would simply cut off the high-*Q* part of the data without otherwise affecting the data quality of the remainder. The line shape is distinctly asymmetric. It has its origin in the spallation neutron pulse shape and will be discussed in the next section.

## 3. Peak shape

**Figure 10:**
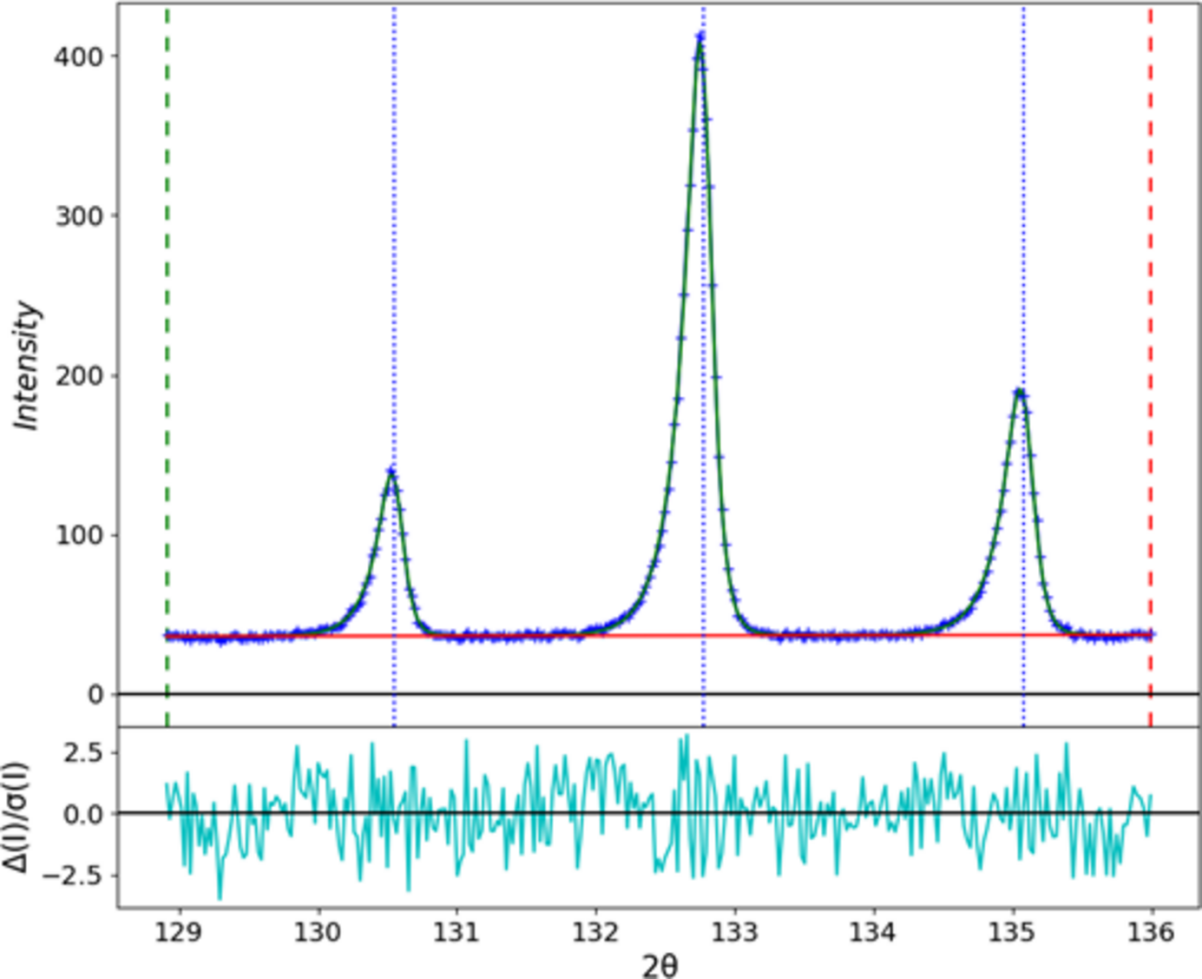
Single-peak fits for three high-angle reflections in the CW POWGEN NIST SRM 660c La^11^B_6_ pattern. Vertical blue dotted lines are the refined peak positions, the blue curve is the observed data and the green curve is the calculated fit; the background (red) and difference divided by error (cyan) are also shown.

The peak-shape asymmetry is similar to what is obtained for conventional neutron TOF powder diffraction (Von Dreele *et al.*, 1982 ▸) and for ‘pink’ beam CW X-ray diffraction (Von Dreele *et al.*, 2021 ▸). Following the latter work, we obtain a function as a Gaussian convoluted with the paired back-to-back exponentials:
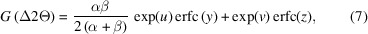
where erfc is the complementary error function,
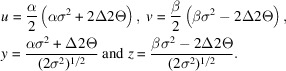
The corresponding Lorentzian convolution is
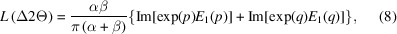
where Im is the imaginary part, *E*_1_ is the exponential integral function,
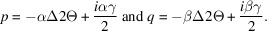
These are combined to give the overall line shape as a pseudo-Voigt with the formulation of η according to Thompson *et al.* (1987 ▸) to closely approximate the Voigt function:
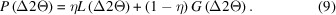
The term Δ2Θ is the offset between a profile point and the calculated 2Θ for the Bragg peak. Thus, this function requires the rise and fall exponential coefficients (α and β) and the Gaussian and Lorentzian coefficients (σ and γ), along with position and intensity. The success of this function can be seen in Fig. 10 ▸, which shows the essentially perfect fit to three high-angle peaks in the La^11^B_6_ pattern; the remaining differences are entirely statistical noise. The peak positions do not coincide with the peak maxima; this is a consequence of the underlying asymmetry of the spallation source emission profile.

Single-peak fits to the entire La^11^B_6_ pattern established the relations for α and β as
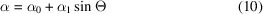
and
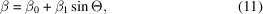
while, as expected for CW data, σ and γ follow the Caglioti *et al.* (1958 ▸) formalism,
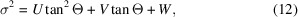
and that used for conventional CW data,
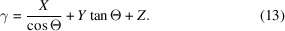
For the convenience of comparing the coefficients with those obtained on conventional CW instruments, the *UVW* coefficients are scaled by 10^4^ and the *XY* coefficients are scaled by 100 within *GSAS-II*; this makes the units for these parameters centidegree squared and centidegree, respectively. These terms are then further modified by the corresponding crystallite size and powder microstrain parameterization within *GSAS-II*, permitting potential extraction of these data from a Rietveld refinement.

The peak positions in Debye–Scherrer CW diffraction patterns are sensitive to placement of the sample relative to the instrument centre (Scarlett *et al.*, 2011 ▸). In *GSAS-II*, this is modelled using the small-angle approximation (α = sin α, in radians) by
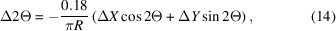
where 2Θ is that calculated from the *d* spacing and wavelength, and *R* is the sample-to-detector distance in millimetres; this gives Δ*X* (in the horizontal plane, perpendicular to the incident beam) and Δ*Y* (parallel to the incident beam, away from the source) in micrometres. (This profile function is implemented in *GSAS-II* for the CW data type ‘PNB’ to distinguish it from the conventional CW type ‘PNC’; see Section S2 for details.)

## 4. Examples

**Figure 11:**
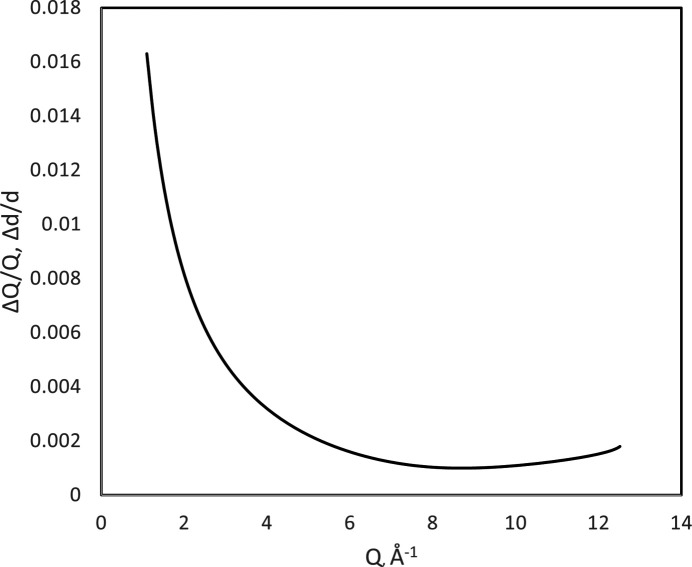
The resolution of CW data from the 1.0 < λ < 2.0 Å band from POWGEN with assigned λ = 1.0 Å based on calibration by NIST SRM 660c La^11^B_6_.

To calibrate POWGEN in the CW mode for the 1 < λ < 2 Å frame, we use the NIST SRM 660c (2015 ▸) La^11^B_6_ pattern shown in Fig. 8 ▸. The SRM certificate gives *a* = 4.156826 Å at 22.5°C for the lattice parameter of this LaB_6_ sample and gives a crystallite size of ∼0.8 µm; there was no evidence of microstrain. With this information, calibration gave coefficients (Table 1 ▸ and Section S2) that describe a good high-resolution (best Δ*d*/*d* ≃ 0.001 at *Q* ≃ 8.0 Å^−1^) CW neutron powder diffractometer (Fig. 11 ▸). There was no evidence of any Lorentzian contribution to the instrumental broadening, and thus the *X*, *Y* and *Z* coefficients are zero. There is also an apparent sample displacement of Δ*Y* = 3669 (16) µm parallel to the incident beam, away from the source. The instrument radius is assigned the mean (3180 mm) for use in equation (14); there was no perpendicular displacement (Δ*X*) since events from both sides of POWGEN were summed together.

**Figure 12:**
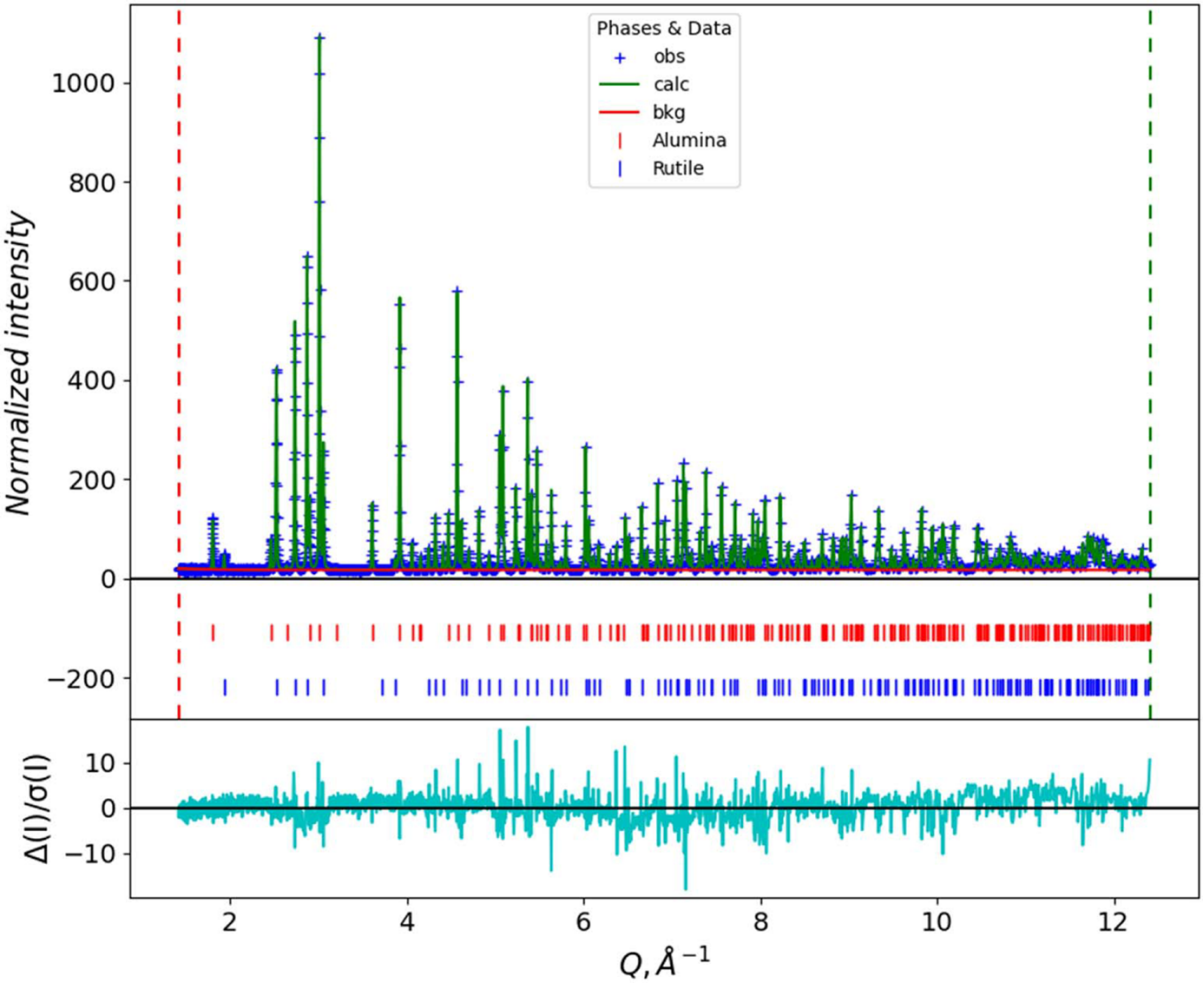
A Rietveld refinement fit for POWGEN TOF data for a selected TiO_2_/Al_2_O_3_ nominal 50/50 by weight NIST SRM 674 certification sample. Items are as shown in the legend; the cyan line below is *I*_obs_ − *I*_calc_ in units of standard error in *I*_obs_.

As a direct comparison between the conventional POWGEN TOF data and new POWGEN CW data extracted from the same suite of event data, we chose one of the samples (a TiO_2_/Al_2_O_3_ mixture nominally 50/50 by weight) used for a NIST SRM recertification trial for SRM 674 (*X-ray Powder Diffraction Intensity Set for Quantitative Analysis by X-ray Powder Diffraction*). The TOF pattern was processed following the conventional POWGEN protocols via equation (1) for data taken in the 1 < λ < 2 Å frame with Δ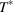/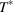 = 0.0004 steps, 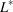 = 63.81 m and 2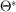 = 90°, and normalized by a vanadium run processed in the same way. The CW pattern was processed as described above, assigning a nominal wavelength of 1.0 Å. Both were subject to Rietveld refinements using *GSAS-II*; instrumental parameters for TOF were used as provided by the SNS facility, while those obtained from La^11^B_6_ described above (Table 1 ▸) were used for the CW data. The fits are shown as a function of *Q* in Figs. 12 ▸ and 13 ▸ to allow easy comparison; numerical results are shown in Table 2 ▸.

Comparing the two datasets, the CW dataset comprises more than 4× more points than the TOF dataset to cover the same *Q* range, and these are more evenly distributed than the TOF data (*cf*. Figs. 2 ▸ and 9 ▸). Consequently, the CW fit has a slightly higher residual. The larger number of points at higher *Q* results in only a slight reduction in the estimated standard errors for most parameters, despite there being as few as three or four points across the entire Bragg peak at the highest *Q* for the TOF data while there will be 10 or more across the CW FWHM for these same peaks. Both datasets were created from the same suite of neutron events. The lattice parameters obtained from the CW data are particularly close to the previous certified values for TiO_2_ and Al_2_O_3_ (*cf*. Table 2 ▸); any difference may be because the materials used here are freshly obtained and not identical to the older SRM materials. Moreover, the POWGEN ambient temperature (∼27°C) is ∼4.6°C higher than the NIST SRM measurement conditions (22.5°C), although this is partially offset by the La^11^B_6_ calibration being run at the same temperature. The sample displacement Δ*Y* = 3764 (14) µm is very similar to that found with the La^11^B_6_ calibration run; this may be a real effect. It is not clear where this offset (∼3.8 mm) comes from since it is present in both the calibration and sample fits; it may have originated in the initial diamond powder run used to calibrate the pixel *K* (= *L* sin Θ) factors (Huq *et al.*, 2019 ▸). Alternatively, it may be a manifestation of the offset in observed TOF due to the electronic *T*_0_; the relative change in flight path (Δ*Y* = 3.7 mm versus 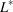 = 63.81 m) is similar to the relative change in TOF (*T*_0_ = 1.51 µs versus 16 < *T* < 32 ms; the true TOF for these POWGEN experiments). The TOF analysis has no correction for sample displacement; refinement of DifA (a diffractometer constant) was included here as an attempt at a second-order correction on the conversion between TOF and *d* spacing. It did not give lattice parameters as close as those from the CW data.

The atomic coordinates found from the two analyses match, but the thermal motion parameters from the CW fit are roughly twice those from the TOF fit. *Mantid* provides a method, *CarpenterSampleCorrection* (Mildner & Carpenter, 1990 ▸), for absorption/multiple scattering corrections for a cylindrical sample; a test application of this on the vanadium spectrum and its subsequent use for the TiO_2_/Al_2_O_3_ sample run gave a new pattern that was essentially the same as that used here, apart from a different scaling factor. The slight shifts in atom thermal motion parameters were all less than their respective estimated standard errors and did not make up the difference between *U*_*ij*_ values obtained from TOF and CW representations of the neutron event data. Thus, it would appear that the absorption correction to the vanadium spectrum is in essence a scaling factor and could be ignored; the sample absorption here is expected to be smaller and presumed to be just a scaling factor as well. In any event, the *U*_*ij*_ values obtained here with the CW data agree quite well with measurements by others (*cf*. Table 2 ▸), especially for TiO_2_.

The values obtained for crystallite size and microstrain are similar for the two data presentations; they indicate that the materials are fine grained and unusually well annealed. Their determinations were facilitated by the good POWGEN instrumental resolution that is free of any apparent Lorentzian broadening [*X* = *Y* = *Z* = 0 in equation (13)].

## 5. Conclusions

Formulating the neutron event data from POWGEN as a CW pattern with pseudo-2Θ instead of a TOF pattern with pseudo-*T* gives data that are easily used in a Rietveld refinement. It can cover a substantial *Q* range (1.0 < *Q* < 12.5 Å^−1^ or 0.5 < *d* < 6.3 Å) in a 10 < 2Θ < 170° scan for the 1 < λ < 2 Å frame in well distributed steps. A different POWGEN data frame will yield a different *Q* range for the same 2Θ scan. The spallation neutron CW powder profile developed here requires fewer instrumental parameters (8) than the corresponding POWGEN TOF profile (12 parameters; Huq *et al.*, 2019 ▸) and easily retains the POWGEN resolution performance at high *Q* (Δ*Q*/*Q* ≃ 0.001 at *Q* ≃ 8.0 Å^−1^), which is desired for crystal structure analyses. Moreover, the coefficients for this CW profile function follow functional forms [equations (10)–(13)] more grounded in the instrument design characteristics than the empirical power series forms (Von Dreele *et al.*, 1982 ▸; Huq *et al.*, 2019 ▸) used for TOF patterns. It also includes an explicit description for sample displacement effects [equation (14)], which are commonly encountered with use of various sample-environment devices (*e.g.* sample changers, furnaces and cryostats); the TOF profile representation has no explicit sample-position parameterization.

Furthermore, the detailed scattering geometry of a given Bragg reflection is still somewhat obscured for the CW data, but the assigned 2Θ is within the possible scattering-angle bounds. For example, the *d* = 1.5 Å Bragg reflection noted above is assigned a scattering angle of ∼39° 2Θ for λ = 1.0 Å, but it is still accumulated from neutron events over a range 39 < 2Θ < 84°. Moreover, the events for this Bragg reflection span a range of azimuth angles from 0 (in the horizontal instrument plane) to about ±22° above and below the instrument plane on one side and ±7° on the other [*cf*. Fig. 2 of Huq *et al.* (2019 ▸)], thus making texture corrections problematic. However, full texture analysis via spherical harmonics analysis (Von Dreele, 1997 ▸) could be performed by fitting POWGEN with a sample goniometer and using only the detector pixels from the 24 detector panels in the horizontal plane to form two CW patterns (12 panels from the right side and 12 panels from the left side of POWGEN). Using only the central band of detector panels limits the azimuthal spread, thus giving data usable for texture analysis. These would have to be accompanied with vanadium and calibration runs partitioned in the same way.

Since the actual wavelength band is rather narrow (*e.g.* 1 < λ < 2 Å), the absorption correction currently employed for CW data in *GSAS-II* (Lobanov & Alte da Veiga, 1998 ▸) will suffice; however, a slight improvement could be made by using the methods in *Mantid* to correct the vanadium and CW patterns for absorption. Finally, extinction will be largely invisible because the true wavelength (at least in the 1 < λ < 2 Å frame) is too short to create much of an effect; the correction is proportional to λ^2^ (Sabine *et al.*, 1988 ▸) and thus is, for example, <2% for reflection intensities from 25 µm silicon grains.

Lastly, new high-resolution powder diffractometers being built at high-power spallation sources can use this approach to make usable CW patterns if the detector layout offers continuous wide-angle coverage over a smooth curve. A long flight path equipped with a supermirror guide, *T*_0_ and frame choppers could provide a peak resolution Δ*d*/*d* < 0.001 over a wide *Q* range well suited for crystal structure analysis.

## Supplementary Material

Supporting information. DOI: 10.1107/S1600576724008756/in5102sup1.pdf

## Figures and Tables

**Figure 13:**
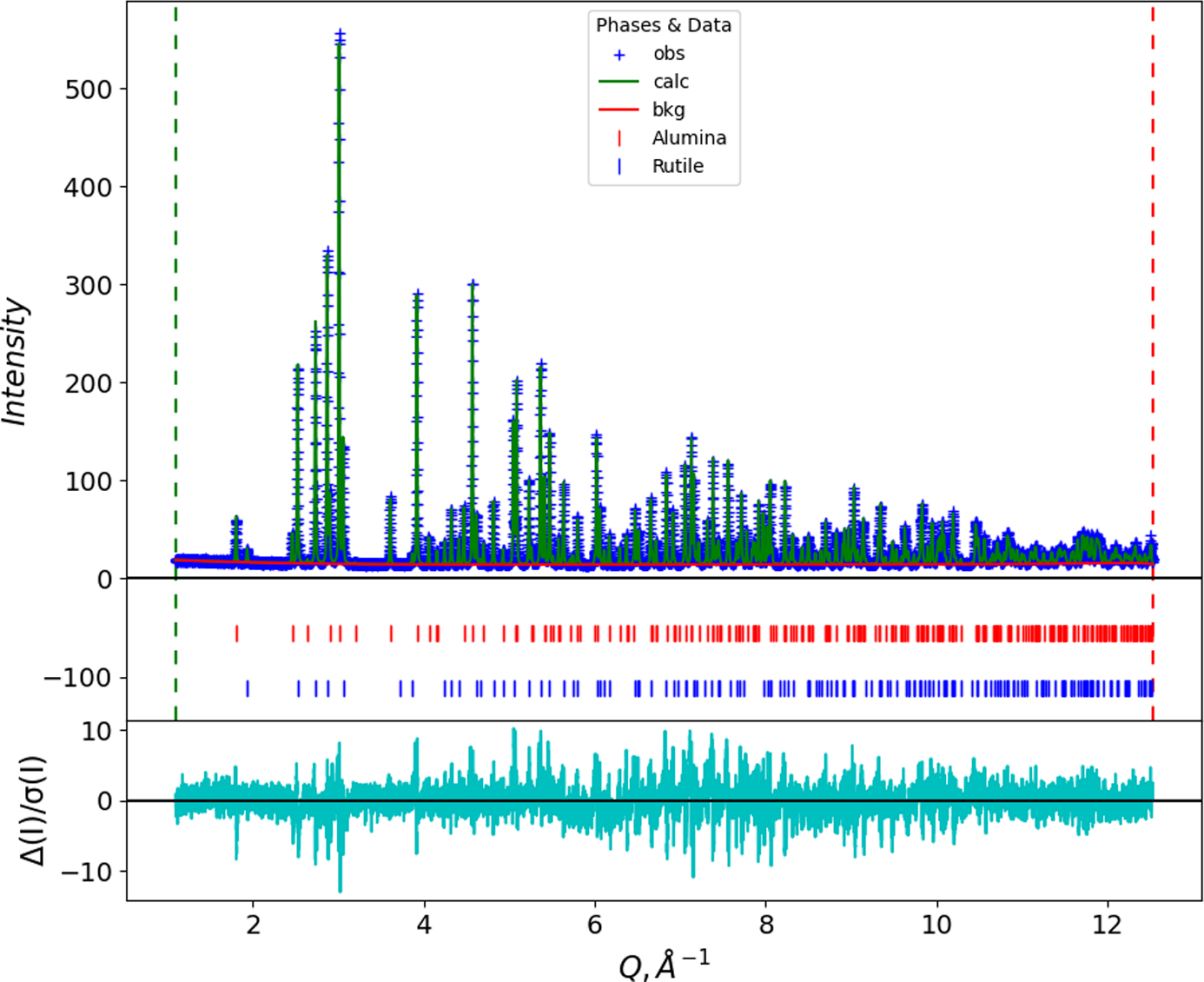
A Rietveld refinement fit for POWGEN CW data for a selected TiO_2_/Al_2_O_3_ nominal 50/50 by weight NIST SRM 674 certification sample. Items are as shown in the legend; the cyan line below is *I*_obs_ − *I*_calc_ in units of standard error in *I*_obs_.

**Table 1:** Instrument parameters [equations (10)–(13)] for CW data from the 1.0 < λ < 2.0 Å band from POWGEN with assigned λ = 1.0 Å based on calibration by NIST SRM 660c La^11^B_6_ Values in parentheses are estimated standard errors from the fit; *R*_wp_ = 5.59% for 11 424 observations.

Wavelength (Å)	1.000568 (4)
*U*	20.85 (33)
*V*	−29.6 (5)
*W*	14.83 (16)
*X*	0
*Y*	0
*Z*	0
α_0_	55.10 (7)
α_1_	−52.15 (3)
β_0_	33.6 (4)
β_1_	28.4 (10)

**Table 2:** Results of Rietveld refinement of TOF and CW data derived from neutron event data for a selected TiO_2_/Al_2_O_3_ nominal 50/50 by weight NIST SRM 674 certification sample Values in parentheses are estimated standard errors obtained from the refinements.

Parameter	TOF	CW	Literature†
*N*_obs_	2715	11441	–
*N*_parameters_	29	32	–
*R*_wp_ (%)	3.2	4.37	–
GOF	2.58	2.18	–
*N*_back_	3	6	–
TiO_2_, *a* (Å)	4.595724 (22)	4.594404 (22)	4.59393 (4)
TiO_2_, *c* (Å)	2.959689 (16)	2.958805 (15)	2.95888 (3)
TiO_2_, O(*x*)	0.30478 (6)	0.30471 (5)	0.30478 (6)
TiO_2_, Ti(*U*_11_)	0.00550 (21)	0.00681 (18)	0.0068 (3)
TiO_2_, Ti(*U*_33_)	0.00345 (33)	0.00466 (27)	0.0046 (5)
TiO_2_, Ti(*U*_12_)	−0.0003 (5)	0.0000 (4)	−0.0004 (3)
TiO_2_, O(*U*_11_)	0.00431 (9)	0.00578 (7)	0.0052 (1)
TiO_2_, O(*U*_33_)	0.00276 (12)	0.00422 (10)	0.0035 (2)
TiO_2_, O(*U*_12_)	−0.00212 (29)	−0.00204 (24)	−0.0020 (2)
Al_2_O_3_, *a* (Å)	4.76179 (4)	4.76019 (4)	4.75936 (8)
Al_2_O_3_, *c* (Å)	12.99889 (6)	12.99443 (6)	12.99231 (15)
Al_2_O_3_, Al(*z*)	0.35210 (4)	0.35215 (4)	0.35216 (1)
Al_2_O_3_, O(*x*)	0.30635 (6)	0.30646 (5)	0.30624 (4)
Al_2_O_3_, Al(*U*_11_)	0.00185 (22)	0.00370 (18)	0.00279 (3)
Al_2_O_3_, Al(*U*_33_)	0.00240 (23)	0.00427 (20)	0.00296 (3)
Al_2_O_3_, O(*U*_11_)	0.00211 (11)	0.00399 (10)	0.00327 (3)
Al_2_O_3_, O(*U*_22_)	0.00221 (11)	0.00416 (10)	0.00341 (3)
Al_2_O_3_, O(*U*_33_)	0.00275 (9)	0.00475 (8)	0.00365 (3)
Al_2_O_3_, O(*U*_13_)	0.0002 (4)	−0.0003 (3)	0.00047 (2)
TiO_2_, weight fraction	0.5007 (10)	0.4981 (9)	–
TiO_2_, size (µm)	0.14630 (27)	0.14156 (20)	–
TiO_2_, microstrain	423 (14)	184 (22)	–
Al_2_O_3_, size (µm)	0.1841 (4)	0.15720 (22)	–
Al_2_O_3_, microstrain	465 (13)	70 (18)	–
DifA	−2.83 (6)	–	–
Sample Δ*Y* (µm)	–	3764 (14)	–

† Rutile (TiO_2_) lattice parameters from NIST SRM 674b (2005 ▸) and atom parameters from Howard *et al.* (1991 ▸). Alumina (Al_2_O_3_) lattice parameters from NIST SRM 676a (2015 ▸) and atom parameters from Lewis *et al.* (1982 ▸).
