# Supplementary material for: Electronic angle focusing for neutron time-of-flight powder diffractometers
Source: J Appl Crystallogr. 2024 Oct 1;57(Pt 5):1588–97. doi: 10.1107/S1600576724008756 (PMC11460386; doi:10.1107/S1600576724008756)
Supplement: Supplementary file 1 [file j-57-01588-sup1.pdf]

JOURNAL OF
APPLIED
CRYSTALLOGRAPHY

Volume 57 (2024)

Supporting information for article:

Electronic angle focusing for neutron time-of-flight powder diffractometers

Robert B. Von Dreele

S1. Preparation of constant wavelength vanadium spectrum

The preparation of the vanadium spectrum is achieved in *Mantid* via the steps in Figure S1. The vanadium event data must be collected under the same operational conditions (*e.g.* wavelength span and detector configuration) for any calibration and sample runs that would need this vanadium normalization. For step #3 in this figure, each event in the loaded file has a TOF and a detector pixel tag. The latter is used to get the instrument pixel geometry factor, $K = 252.778L\sin\Theta/\pi$, needed to convert TOF to Q via a combination of Bragg's Law and the de Broglie equation. In step #5, the *Rebin* method distributes the Q based events into 43120 spectra, one for each detector pixel, covering the approximate range 0.3Å^{-1} < Q < 12.9Å^{-1} in ΔQ=0.001Å^{-1} steps; this must match the result for *Rebin* on any sample data that uses this vanadium pattern for normalization. These are summed in step #7 to produce a single vanadium powder pattern. Because the Mantid method, *StripVanadiumPeaks*. only operates in d-spacing, step #8 converts the Q bin values to d-spacing. Step #9 strips the vanadium peaks and step #10 converts the d-spacing bins back to Q. This *Mantid* workspace ("Vqs") is then saved for use in processing sample data collected within the same SNS/POWGEN operational schedule.

```
# 1) import mantid algorithms
import mantid.simpleapi as mas
# 2) load event data for vanadium
ws = mas.Load('PG3_50353.nxs.h5')
# 3) convert events from TOF & pixel parameters to Q
wsq = mas.ConvertUnits("ws","MomentumTransfer")
# 4) clear no longer needed workspaces
mas.DeleteWorkspaces(["ws",])
# 5) form 2D grid as pixel, Q by summing events with ΔQ=0.001Å-1
vwq = mas.Rebin("wsq",0.001)
# 6) clear no longer needed workspaces
mas.DeleteWorkspaces(["wsq",])
# 7) form histogram on Q by summing all pixels with same Q
vwqs = mas.SumSpectra("vwq")
# 8) convert Q to d-spacing
vwd = mas.ConvertUnits("vwqs","dSpacing")
# 9) strip vanadium Bragg peaks
vwsnp = mas.StripVanadiumPeaks("vwd")
# 10) convert d-spacing to Q; this workspace to be saved for normalization
Vqs = mas.ConvertUnits("vwsnp","MomentumTransfer")
```

Figure S1 *Mantid* python script for making constant wavelength vanadium normalization pattern. The steps are: load, conversion to Q, binning on ΔQ=0.001Å^{-1} steps, conversion to d-spacing, vanadium Bragg peak removal, and conversion back to Q. The resulting Mantid workspace ("Vqs") is saved as "Vqs.nxs" for use in vanadium normalization (cf. Figure 3). Each step is commented in detail.

S2. Importing constant wavelength data from POWGEN into *GSAS-II*

The ".qye" file produced by the *Mantid* python script contains a header block and 3 columns of data values: Q, I and E (= σ_I), and is read into GSAS-II via the "from Topas xye/qye…" importer. The default wavelength is λ=1.0 Å for Q to 2Θ conversion; the header must contain a line with

#q λ

to use a different wavelength (e.g., for a different wavelength band). It should be the lowest wavelength in the band to give the widest available Q range from the data.

The associated instrument file (Figure S2) describes the data type ("PNB") and those parameters obtained from calibration with a standard material and describe the profile and some other parameters for constant wavelength POWGEN data.

```
Type:PNB
Bank:1.0
Lam:1.0005676434708675
Azimuth:0.0
Zero:0.0
U:20.848280190142287
V:-29.63705144906882
W:14.831038082638443
X:0.0
Y:0.0
Z:0.0
alpha-0:55.1037775136401
alpha-1:-52.15319579690605
beta-0:33.64612460503642
beta-1:28.367924269847986
```

Figure S2 CW POWGEN instrument parameter file contents for the 1.0Å < λ < 2.0Å band.
